# The association between pancreatic diseases and pancreatic fat content: a cross-sectional study from the UK Biobank

**DOI:** 10.3389/fendo.2025.1591652

**Published:** 2025-06-06

**Authors:** Jing Gao, Xiaowu Dong, Xiaolei Shi, Yuqing Yang, Weiwei Chen, Weiming Xiao, Guotao Lu, Xiaoping Yu

**Affiliations:** ^1^ Pancreatic Center, Department of Gastroenterology, Yangzhou Key Laboratory of Pancreatic Disease, The Affiliated Hospital of Yangzhou University, Yangzhou University, Yangzhou, China; ^2^ School of Nursing, School of Public Health, Yangzhou University, Yangzhou, China; ^3^ Clinical Medical College, Yangzhou University, Yangzhou, China; ^4^ Department of Health Management Center, The Affiliated Hospital of Yangzhou University, Yangzhou University, Yangzhou, China

**Keywords:** intra-pancreatic fat deposition, pancreatic diseases, type 2 diabetes mellitus, UK Biobank, mediation analysis

## Abstract

**Objective:**

Many researches have demonstrated an association between intra-pancreatic fat deposition (IPFD) and several pancreatic pathological conditions, including pancreatitis, pancreatic cancer, and type 2 diabetes mellitus (T2DM). The aim of this study is to investigate the influence of pancreatic diseases on the accumulation of pancreatic fat, to further explore which kind of pancreatic disease is significant, and to find out the possible mediating factors.

**Methods:**

A cross-sectional study based on the UK Biobank (UKB) data categorized participants by pancreatic disease status and collated relevant information. IPFD was measured using MRI in combination with a deep learning-based organ segmentation model, nnUNet. Linear regression models and mediation analysis were employed to explore the association between pancreatic diseases and IPFD.

**Results:**

Among 61,088 participants, those with pancreatic diseases exhibited higher IPFD than those without (pancreatic endocrine diseases: 11.72% *vs* 7.94%, P<0.001; pancreatic exocrine diseases: 9.44% *vs* 8.03%, P<0.001). After adjusting for multiple variables, a positive association between pancreatic endocrine diseases (particularly T2DM) and IPFD persisted, but not for pancreatic exocrine diseases. Obesity and dyslipidemia partially explained the relationship between T2DM and IPFD.

**Conclusion:**

Pancreatic exocrine disorders are not associated with an increased risk of IPFD, whereas pancreatic endocrine disorders, particularly T2DM, may exhibit a positive relationship. However, the possibility of reverse causation cannot be discounted.

## Introduction

The accumulation of adipose tissue in the pancreas is referred to as “intra-pancreatic fat deposition (IPFD)” (1). Fatty pancreas (FP), also termed pancreatic steatosis, pancreatic fat infiltration, or non-alcoholic fatty pancreas disease, is the excessive deposition of fat in pancreatic tissue (2). A threshold of 6.2% for quantification of normal pancreatic fat content has been established in one study (3).

IPFD lacks specific clinical manifestations, but the development of advanced methods for detecting pancreatic fat has provided avenues for understanding the disease, such as ultrasound, computed tomography (CT), and magnetic resonance imaging (MRI). An efficacious methodology for the evaluation of adipose tissue is the utilization of magnetic resonance imaging proton density fat fraction (MRI-PDFF). MRI-PDFF is frequently employed as a biomarker for the quantification of liver fat. This is achieved by quantifying the relative amounts of water and fat signals in tissue through MRI scans of the liver, thereby enabling the accurate and rapid quantification of liver fat content. It is also applicable to pancreatic tissue.

There is a notable relationship between excessive IPFD, or FP, and the development of pancreatic diseases. A large number of studies have shown that IPFD was a significant contributing factor to a series of pancreatic diseases, including but not limited to acute pancreatitis, chronic pancreatitis, pancreatic cancer, and T2DM (4). In a cohort study, FP was proven to be independently associated with the subsequent development of diabetes (5). Makoto Fujii found in his 6-year cohort study that IPFD may be a risk factor for subclinical chronic pancreatitis (6). In addition, the causal role of IPFD in pancreatitis was confirmed by a Mendelian randomization (MR) study, which suggested that reducing fat deposition in the pancreas meant a reduced risk of pancreatitis (7). However, the reverse MR analysis did not support the reverse causal relationship between pancreatitis and IPFD.

High IPFD and FP were demonstrated to be important risk factors for pancreatic endocrine and exocrine diseases. However, whether pancreatic diseases have an impact on pancreatic fat content, there are very few studies on this issue. The objective of this study is to explore the relationship between previous pancreatic diseases and IPFD through a cross-sectional study, utilizing MRI to quantify pancreatic fat content.

## Methods

### Participants

**Figure 1:**
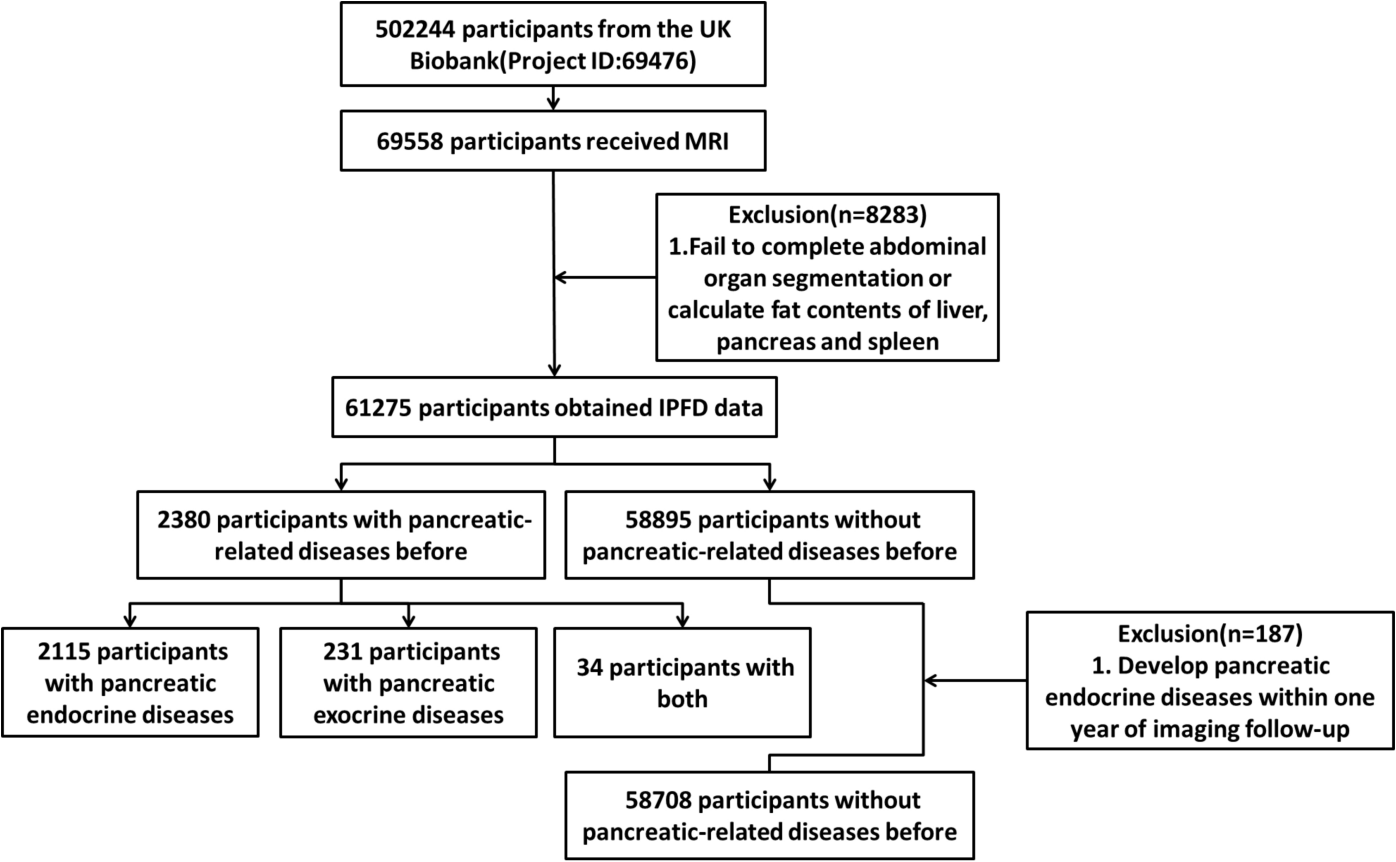
Study design.

The UK Biobank recruited 502,244 participants from the general population. Since the recruitment, researchers have conducted multiple follow-ups, among which 69,558 participants underwent an abdominal Dixon MRI examination during the second follow-up. We followed the methodology of a previous study by our research group and calculated the level of IPFD (8). A total of 61,275 participants from the UK Biobank were therefore included in the analysis, including 2,380 participants with previous pancreatic diseases and 58,895 participants without previous pancreatic diseases. To minimize the risk of reverse causality, a 12-month period free of pancreatic endocrine disease was defined. This excluded participants (n=187) who developed pancreatic endocrine diseases within one year after the imaging follow-up, resulting in a final sample of 58,708 participants without previous pancreatic diseases. (**Figure 1**).

At the outset of the study, a total of 51 pancreatic diseases, according to the International Classification of Diseases, Tenth Revision (ICD-10) (**Supplementary Tables S1**, **S2**), were identified through a search of the UK Biobank diagnosis database which were further classified. There were 2,115 participants with pancreatic endocrine diseases (n=2,115), 231 participants with pancreatic exocrine diseases (n=231), and 34 participants with both pancreatic endocrine and exocrine diseases (n=34) before imaging follow-up (**Figure 1**). During the further screening for pancreatic endocrine disorders, four conditions (C254, E144, E164, and E168) were identified due to their diagnosis post-radiological follow-up. It should be particularly pointed out that in the ICD-10 coding system, E10 denotes type 1 diabetes mellitus, while E11 denotes type 2 diabetes mellitus (9).

The study was approved by the North West Multi-Center Research Ethics Committee, and all participants provided written informed consent. Furthermore, this study was reviewed and approved by the UK Biobank (project ID: 69476).

### Study design

The study was conducted using a cross-sectional design. The clinical characteristics, lifestyle habits, and lipid metabolism situation were compared in the pancreatic endocrine disease group and the pancreatic exocrine disease group separately. Lipid metabolism status (categorical) was defined based on the E78 series of codes in ICD-10. Linear regression models were used to analyze the association between pancreatic diseases and IPFD. The initial model, designated as Model 0, was a single-variable model. Model 1 was adjusted for age, sex (biological), ethnicity, and body mass index (BMI). Model 2 was further adjusted for smoking and drinking status, television watching duration, sleep duration, and weekly exercise duration. In the final model (Model 3), the lipid metabolism situation was additionally adjusted. Multiple linear regression was employed once again for an in-depth analysis of pancreatic endocrine diseases and IPFD.

R Software was used to estimate whether the association between T2DM and IPFD was mediated by obesity (a BMI of 28 or higher) or lipid metabolism situation, using the ‘mediate’ function from the mediation package in R, with the ‘sims’ parameter set to 1,000 to ensure the reproducibility of the results. The mediation effect with its 95%CI was estimated using the bootstrap method and the routes were as follows:

Route 1: T2DM (exposure) → IPFD (outcome).
Route 2: T2DM (exposure) → Obesity or dyslipidemia (mediator) → IPFD (outcome).

It should be mentioned that, like most clinical studies, this study had missing data, and the missing observations might affect the accuracy and reliability of the analysis results. To address this issue, we used the random forest imputation method to estimate missing values. This method predicts and imputes missing values by constructing multiple decision trees and leveraging the information of other variables in the dataset. In comparison to traditional interpolation techniques, it is capable of capturing complex relationships among variables more effectively, thereby enhancing the accuracy of interpolation. We implemented this approach using the R package `mice` with the following code: `micedata <- mice(data_Y, m=5, maxit=50, method=“rf”, seed=500)`, which generated five imputed datasets. Subsequently, we selected the dataset with the lowest AIC and BIC values from the generated datasets for further analysis (10). This selection criterion ensures optimal model fit and prediction accuracy. Before imputing data, we conducted statistical tests to ensure that there were no significant differences in the distribution of data before and after imputation.

### Statistical analysis

Continuous variables were expressed as median (interquartile range). Categorical variables were expressed as n (percentage). Non-parametric tests for continuous variables were used to compare basic characteristics and differences between groups. Chi-square tests were used to analyze categorical variables. We extracted the data through SAS and performed data cleaning and statistical analysis in R version 4.3.2. A p-value of less than 0.05 (two-sided) was considered statistically significant.

### Sensitivity analysis

In the **Supplementary Materials**, we conducted a sensitivity analysis. The method of deleting missing data was used to handle missing observations for we wanted to avoid the potential bias introduced by imputation. After deleting data, the sample size was reduced from 61,088 to 47,292, but we observed that the overall characteristics of the data, such as median and interquartile range, remained relatively stable. We then conducted baseline analysis, model adjustment, and mediation analysis as well.

## Results

### Characteristics of the participants

**Table 1** summarized the baseline characteristics of individuals with and without pancreatic diseases (**Table 1**). Individuals with pancreatic endocrine diseases were observed to be older, more frequently male white, and exhibited higher BMI values compared to those without such diseases. There were also significant differences in smoking or drinking status and lipid metabolism situation (P<0.001). Furthermore, individuals with the diseases exhibited increased sedentary time and reduced weekly physical activity.

**Table 1:** The baseline characteristics of participants.

Characteristics	Overall	Pancreatic endocrine diseases		P	Pancreatic exocrine diseases		P
	(N=61088)	No(N=58939)	Yes(N=2149)		No(N=60823)	Yes(N=265)	
Age	66.00 [59.00, 71.00]	65.00 [59.00, 71.00]	70.00 [64.00, 74.00]	<0.001	66.00 [59.00, 71.00]	69.00 [63.00, 74.00]	<0.001
Sex				<0.001			0.331
Female	31672 (51.8)	30914 (52.5)	758 (35.3)		31543 (51.9)	129 (48.7)	
Male	29416 (48.2)	28025 (47.5)	1391 (64.7)		29280 (48.1)	136 (51.3)	
Ethnic background				<0.001			0.986
Others	1971 (3.2)	1795 (3.0)	176 (8.2)		1963 (3.2)	8 (3.0)	
White	59117 (96.8)	57144 (97.0)	1973 (91.8)		58860 (96.8)	257 (97.0)	
BMI	25.99 [23.54, 28.95]	25.91 [23.49, 28.82]	28.78 [25.66, 32.32]	<0.001	25.98 [23.54, 28.94]	27.26 [24.38, 31.05]	<0.001
Smoking				<0.001			0.704
Never	38195 (62.5)	37083 (62.9)	1112 (51.7)		38035 (62.5)	160 (60.4)	
Previous	20890 (34.2)	19935 (33.8)	955 (44.4)		20793 (34.2)	97 (36.6)	
Current	2003 (3.3)	1921 (3.3)	82 (3.8)		1995 (3.3)	8 (3.0)	
Alcohol				<0.001			<0.001
Never	2040 (3.3)	1909 (3.2)	131 (6.1)		2020 (3.3)	20 (7.5)	
Previous	2246 (3.7)	2106 (3.6)	140 (6.5)		2218 (3.6)	28 (10.6)	
Current	56802 (93.0)	54924 (93.2)	1878 (87.4)		56585 (93.0)	217 (81.9)	
Time spent watching television (TV)	3.00 [2.00, 4.00]	3.00 [2.00, 4.00]	3.00 [2.00, 5.00]	<0.001	3.00 [2.00, 4.00]	3.00 [2.00, 4.00]	<0.001
Sleep duration	7.00 [7.00, 8.00]	7.00 [7.00, 8.00]	7.00 [6.00, 8.00]	0.239	7.00 [7.00, 8.00]	7.00 [7.00, 8.00]	0.559
Summed MET minutes per week for all activity	2190.00 [1132.50, 3912.00]	2213.00 [1150.00, 3932.00]	1704.00 [792.00, 3439.50]	<0.001	2190.00 [1133.00, 3916.25]	2186.00 [924.00, 3732.00]	0.193
Dyslipidemia				<0.001			<0.001
No	56282 (92.1)	54876 (93.1)	1406 (65.4)		56067 (92.2)	215 (81.1)	
Yes	4806 (7.9)	4063 (6.9)	743 (34.6)		4756 (7.8)	50 (18.9)	
IPFD	8.03 [6.34, 11.49]	7.94 [6.30, 11.29]	11.72 [8.39, 18.32]	<0.001	8.03 [6.33, 11.48]	9.44 [7.09, 13.94]	<0.001

Continuous values were presented as median (interquartile range) and categorical variables were presented as counts (percentages).

BMI, Body mass index; MET, Metabolic equivalent task; IPFD, Intra-pancreatic fat deposition.

When analyzing the characteristics of individuals with and without pancreatic exocrine diseases, the results revealed that those with the disease also showed relatively older age and higher BMI values. There were significant differences in drinking status and lipid metabolism situation as well (P<0.001).

### Associations of IPFD with pancreatic diseases

Through the table and vioplot (**Table 1**, **Supplementary Figure S1**), we found that the pancreatic fat content of individuals with pancreatic diseases was significantly higher than those without, whether it was pancreatic endocrine diseases or pancreatic exocrine diseases (pancreatic endocrine diseases: 11.72% *vs* 7.94%, P<0.001; pancreatic exocrine diseases: 9.44% *vs* 8.03%, P<0.001).

### Linear regression analysis

In the UKB, the linear regression model 0 demonstrated a positive association between pancreatic endocrine diseases and IPFD (regression coefficient (β) = 4.76; 95% confidence interval (CI): 4.47-5.06; P<0.001). After adjusting for covariates, the association between pancreatic endocrine diseases and IPFD still existed. Upon the inclusion of additional blood lipid factors in the linear regression model, the regression coefficient associated with pancreatic endocrine diseases also underwent a change (regression coefficient (β) =1.86; 95% confidence interval (CI): 1.60-2.13; P<0.001) (**Table 2**).

**Table 2:** The extent to which pancreatic diseases alone and in combination with traditional independent variables, lifestyle habits and dyslipidemia affect IPFD.

Models	Pancreatic endocrine diseases		Pancreatic exocrine diseases	
	β (95%CI)	P	β (95%CI)	P
Model 0	4.76 (4.47, 5.06)	<0.001	1.89 (1.05, 2.72)	<0.001
Model 1	2.13 (1.86, 2.39)	<0.001	0.36 (-0.37, 1.10)	0.334
Model 2	2.02 (1.76, 2.29)	<0.001	0.27 (-0.47, 1.00)	0.478
Model 3	1.86 (1.60, 2.13)	<0.001	0.20 (-0.54, 0.93)	0.599

Model 0: unadjusted covariates.

Model 1: adjusted for age, sex, ethnic background and BMI.

Model 2: adjusted for age, sex, ethnic background, BMI, smoking status, alcohol drinker status, time spent watching television (TV), sleep duration and summed MET minutes per week for all activity.

Model 3: adjusted for age, sex, ethnic background, BMI, smoking status, alcohol drinker status, time spent watching television (TV), sleep duration, summed MET minutes per week for all activity and dyslipidemia.

IPFD, Intra-pancreatic fat deposition; BMI, Body mass index; MET, Metabolic equivalent task.

In contrast, pancreatic exocrine diseases in the linear regression model were only related to IPFD when they existed as a standalone condition (regression coefficient (β) =1.89; 95% confidence interval (CI): 1.05-2.72; P<0.001). Following adjustment for other variables, the results were no longer statistically significant (**Table 2**).

In order to further explore the relationship between pancreatic endocrine diseases and IPFD, we also employed multiple linear regression for analysis. After adjusting for potential confounding factors, the results indicated a significant association between T2DM and IPFD (regression coefficient (β) =2.15; 95% confidence interval (CI): 1.87-2.43; P<0.001), as opposed to type 1 diabetes mellitus (T1DM) (regression coefficient (β) =-0.62; 95% confidence interval (CI): -1.48-0.25; P=0.165) (**Supplementary Table S3**).

### Mediation analysis

**Figure 2:**
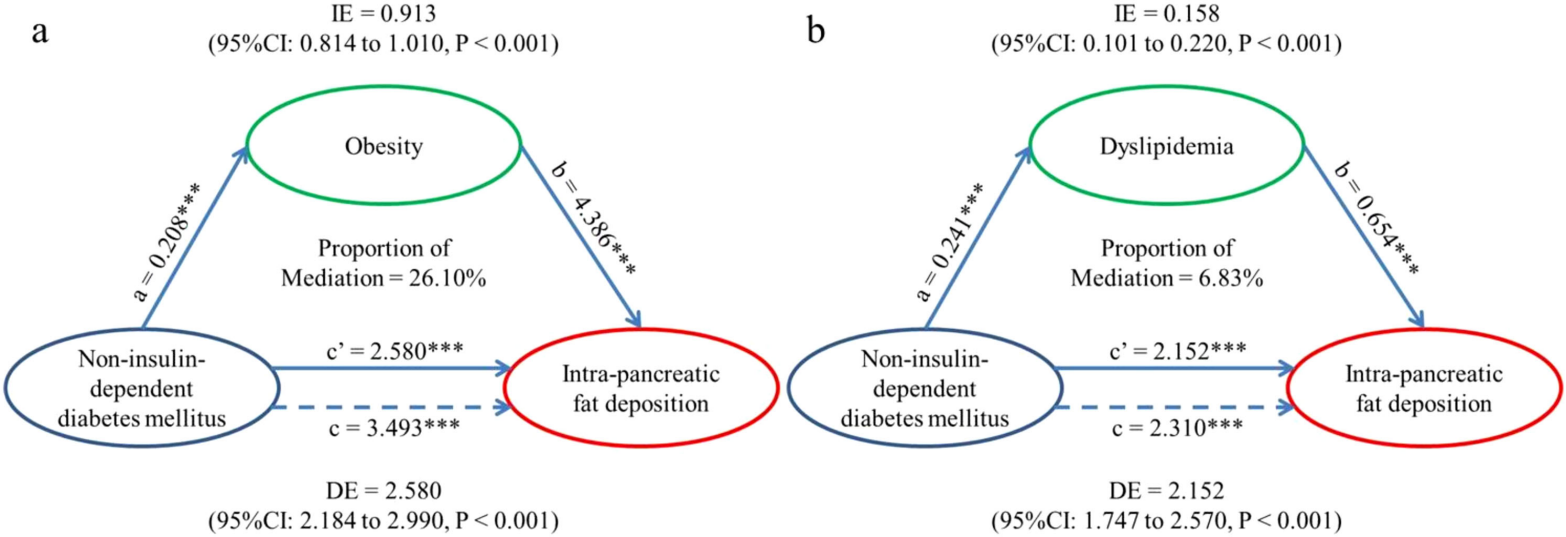
Mediation models. **(a)** Indirect effect (0.913; P < 0.001) of T2DM (exposure) towards IPFD (outcome) which was transmitted through obesity (mediator). Direct effect (2.580; P < 0.001) of T2DM (exposure) towards IPFD (outcome) which was the residual influence after accounting for obesity (mediator). Total effect (3.493; P < 0.001) of T2DM (exposure) towards IPFD (outcome) without considering the effect of obesity. **(b)** Indirect effect (0.158; P < 0.001) of T2DM (exposure) towards IPFD (outcome) which was transmitted through dyslipidemia (mediator). Direct effect (2.152; P < 0.001) of T2DM (exposure) towards IPFD (outcome) which was the residual influence after accounting for dyslipidemia (mediator). Total effect (2.310; P < 0.001) of T2DM (exposure) towards IPFD (outcome) without considering the effect of dyslipidemia. IPFD, Intra-pancreatic fat deposition; IE, Indirect effect, equivalent to a*b; DE, Direct effect, equivalent to c’; TE, Total effect, equivalent to c. ∗∗∗P, < 0.001.

In light of the strong association between T2DM and IPFD, mediation analysis (**Figure 2**) was performed to examine whether the association could be explained by obesity or dyslipidemia while adjusting for confounders. The results showed that the association was mediated by obesity (indirect coefficient (95%CI): 0.913(0.814-1.010), P<0.001) and dyslipidemia (indirect coefficient (95%CI): 0.158(0.101-0.220), P<0.001) in the UKB dataset. And the proportion of mediation was different: 26.10% of the association could be explained by obesity and only 6.83% by dyslipidemia.

### Sensitivity analysis

Through sensitivity analysis (**Supplementary Tables S4**-**S6**, **Supplementary Figures S2**, **S3**), we observed that the impact of parameter changes on the results was negligible, indicating a high degree of consistency in our research outcomes.

## Discussion

The study revealed a significant relationship between pancreatic diseases and pancreatic fat content. In particular, individuals with a history of pancreatic endocrine diseases (T2DM) might exhibit elevated pancreatic fat content in comparison to others.

In the academic community, two main perspectives existed regarding the relationship between pancreatic diseases and IPFD. One perspective posited that IPFD was a direct consequence of pancreatic disease development. The opposing view was based on PANDORA’s theory (11), which proposed an inverse causal chain, highlighting fatty pancreas as a mechanistic driver of most non-genetic pancreatic diseases. Our team previously validated the scientific merit of PANDORA’s theory through experimentation (8). We now aim to delve deeply into the hypothesis that IPFD is a consequence of pancreatic diseases, exploring the first perspective in greater detail.

After adjusting for variables, only pancreatic endocrine diseases were related to an increased risk of IPFD. We similarly utilized multiple linear regression analysis to dig into the finding which indicated a significant association between T2DM and IPFD. The pancreatic fat content of men with T2DM and non-diabetic men was assessed by Tushuizen, and the results showed that the average pancreatic fat content of diabetic patients was 20.4% compared to 9.7% in the control group (12). T2DM was regarded as a significant contributing factor in the pathogenesis of nonalcoholic fatty liver disease (NAFLD) (13). Meanwhile, Juyeon noted that IPFD and liver fat were interrelated, and used mediation analysis to demonstrate that the effect of liver fat on IPFD was both direct and indirect (14). So there was reason to believe that T2DM would increase the risk of IPFD. From another point of view, Patel et al. advanced the proposition that a fatty pancreas could result from a condition of insulin resistance, a hallmark feature of T2DM (15). Furthermore, it was demonstrated that hyperglycemia gave rise to a sequence of enzymatic reactions, which in turn resulted in the inhibition of mitochondrial β-oxidation in pancreatic β-cells, ultimately leading to the accumulation of intracellular triglycerides (16). Taken together, these findings favored the view of IPFD as a consequence of pancreatic endocrine diseases, not just a contributing factor.

In T2DM with insulin resistance, the body secretes more insulin (17), which hinders the body’s ability to break down fat (18), to regulate blood glucose levels. Researches have also shown that pharmacologic reduction of insulin would alleviate hyperphagia and weight gain in a variety of species (17). On the other hand, a high BMI would increase the tendency to accumulate ectopic fat in the pancreas and subsequent pancreatic dysfunction (19). Obesity was also positively correlated with FP, consistent with the observations of Wu and Wang (20). Dyslipidemia is typically defined as an elevation in the concentration of lipids (such as cholesterol and triglycerides) in the bloodstream above the normal range, which is often considered to be a key factor contributing to fat deposition in tissues (21). Specifically speaking, Singh noted a positive correlation of IPFD with hypertriglyceridemia and noticed reduced concentrations of high-density lipoprotein cholesterol (HDL-C) as well when reviewing markers of pancreatic fat in blood (22). In short, obesity and dyslipidemia (as components of MetS) were both significant factors in the development of IPFD (23). Considering all the above, it was reasonable to suppose that the relationship between T2DM and IPFD could be explained by obesity and dyslipidemia and our findings in the mediation analysis supported the assumption: obesity and dyslipidemia mediated the association, although the effects were different (26.10% of the association explained by obesity and only 6.83% explained by dyslipidemia). Given that the proportions were relatively modest, it was necessary to further explore the mechanisms involved to better explain the association. However, some observational studies conducted on non-diabetic obese individuals using ultrasonography have indicated that pancreatic fat accumulation may not always be significantly associated with systemic insulin resistance (24).

Some researchers hypothesized that the pathophysiology related to pancreatic fat deposition was pancreatic acinar cell death and replacement by fat cells which were caused by pancreatic-related diseases, such as pancreatic duct obstruction (chronic obstructive pancreatitis) (25). However, in PANDORA’s theory (11), Petrov MS concluded that pancreatic cancer was a result of fatty pancreas, not a promoter, and in our study’s multi-factor analysis, no relationship was found between pancreatic exocrine diseases and IPFD as well. The preliminary finding was intriguing and merited further exploration in subsequent studies.

Additionally, it was estimated that 16% to 35% of the general population would have pancreatic steatosis, depending on race and age (26). Age was identified as an independent risk factor for FP (27). Saisho (28) reported that pancreatic fat content increased with age throughout childhood until it reached a plateau at the age of 50. In a study (29) on sex and IPFD, it was found that men had significantly greater visceral fat deposits than women. Additionally, men exhibited significantly elevated triglyceride levels in their blood. This may be due to hormonal differences between men and women, but the specific mechanism was not clear. What’s more, the study (29) also pointed out the association between IPFD and the pancreatic site. This part of the research was exactly what our study lacked. For smoking and alcohol consumption, they both contributed to IPFD (30). Prolonged television viewing represented a sedentary lifestyle and might be associated with an increased risk of visceral fat accumulation. The finding of our study corroborated that of Sugiura’s study (31).

Prior researches have indicated that an elevation in pancreatic fat content contributed to an increased likelihood of developing pancreatic diseases. A prospective cohort study conducted by our team (8) investigated the association between IPFD and pancreatic endocrine and exocrine diseases and found that excessive IPFD, or FP, was an important risk factor for some pancreatic diseases (AP, PC, and DM). And the results of our study suggested a potential association between previous pancreatic endocrine diseases (T2DM) and an increased risk of IPFD. It can be seen, therefore, that IPFD and pancreatic diseases may interact with each other in a manner that results in the continuous promotion of disease progression. In the clinical management of T2DM, it is crucial to monitor the changes in pancreatic fat content. Clinicians should be vigilant regarding the potential for increased pancreatic fat content to exacerbate insulin resistance and impair islet β-cell function, thereby contributing to the progression of diabetes.

Our study enabled us to gain insight into the characteristics of individuals with pancreatic diseases and to examine the potential association between pancreatic diseases and pancreatic fat content. It should be noted, however, that the present study was not without limitations. Firstly, it was not possible to determine the causal relationship between pancreatic diseases and pancreatic fat content using cross-sectional studies. Secondly, the majority of the data were derived from the white population. Given the considerable genetic heterogeneity among human populations, the findings of this study should be interpreted with caution when extrapolated to other groups. Thirdly, it was not possible to discount the potential influence of unidentified or unquantified confounding factors on the relationship between pancreatic diseases and IPFD. Fourth, this study aimed to explore the association between previously diagnosed pancreatic diseases and pancreatic fat content, but during this data collection process, it was not possible to rule out the possibility that participants had IPFD before the diagnosis of pancreatic diseases (meaning that it may be the case that a participant had IPFD before being diagnosed with pancreatic-related diseases, but the UKB imaging data collection time was later than both the pancreatic disease diagnosis time and IPFD onset time. In this way, in data analysis, it was impossible to determine the causality).

In summary, there is an association between pancreatic diseases and increased pancreatic fat content, with a stronger association observed between T2DM and IPFD. It would be beneficial for future studies to employ longitudinal designs to ascertain the causal relationship.

## Data Availability

The datasets presented in this study can be found in online repositories. The names of the repository/repositories and accession number(s) can be found in the article/**Supplementary Material**.
