# Supplementary material for: The association between pancreatic diseases and pancreatic fat content: a cross-sectional study from the UK Biobank
Source: Front Endocrinol (Lausanne). 2025 Jun 6;16:1591652. doi: 10.3389/fendo.2025.1591652 (PMC12178842; doi:10.3389/fendo.2025.1591652)
Supplement: Supplementary file 1 [file DataSheet1.docx]

**Supplementary materials**

Table S1: Pancreatic endocrine diseases included in the study (ICD-10 coding system, 30 in total)

Table S2: Pancreatic exocrine diseases included in the study (ICD-10 coding system, 21 in total)

Figure S1: Vioplot of IPFD grouped by the history of pancreatic diseases

Table S3: Impact of the five pancreatic endocrine disorders on IPFD separately

Table S4: The baseline characteristics of participants (sensitivity analysis)

Figure S2: Vioplot of IPFD grouped by the history of pancreatic diseases (sensitivity analysis)

Table S5: The extent to which pancreatic diseases alone and in combination with traditional independent variables, lifestyle habits and dyslipidemia affect IPFD (sensitivity analysis)

Table S6: Impact of the five pancreatic endocrine disorders on IPFD separately (sensitivity analysis)

Figure S3: Mediation models (sensitivity analysis)

Table S1: Pancreatic endocrine diseases included in the study (ICD-10 coding system, 30 in total)

| Coding | | Meaning | |
| --- | --- | --- | --- |
| C25 | C254 | Malignant neoplasm of pancreas | Endocrine pancreas |
| E10 | E100 | Insulin-dependent diabetes mellitus | With coma |
|  | E101 |  | With ketoacidosis |
|  | E102 |  | With renal complications |
|  | E103 |  | With ophthalmic complications |
|  | E104 |  | With neurological complications |
|  | E105 |  | With peripheral circulatory complications |
|  | E106 |  | With other specified complications |
|  | E108 |  | With unspecified complications |
|  | E109 |  | Without complications |
| E11 | E110 | Non-insulin-dependent diabetes mellitus | With coma |
|  | E111 |  | With ketoacidosis |
|  | E112 |  | With renal complications |
|  | E113 |  | With ophthalmic complications |
|  | E114 |  | With neurological complications |
|  | E115 |  | With peripheral circulatory complications |
|  | E116 |  | With other specified complications |
|  | E118 |  | With unspecified complications |
|  | E119 |  | Without complications |
| E13 | E136 | Other specified diabetes mellitus | With other specified complications |
|  | E139 |  | Without complications |
| E14 | E141 | Unspecified diabetes mellitus | With ketoacidosis |
|  | E143 |  | With ophthalmic complications |
|  | E144 |  | With neurological complications |
|  | E149 |  | Without complications |
| E16 | E160 | Other disorders of pancreatic internal secretion | Drug-induced hypoglycaemia without coma |
|  | E161 |  | Other hypoglycaemia |
|  | E162 |  | Hypoglycaemia, unspecified |
|  | E164 |  | Abnormal secretion of gastrin |
|  | E168 |  | Other specified disorders of pancreatic internal secretion |

Table S2: Pancreatic exocrine diseases included in the study (ICD-10 coding system, 21 in total)

| Coding | | Meaning | |
| --- | --- | --- | --- |
| C25 | C250 | Malignant neoplasm of pancreas | Head of pancreas |
|  | C251 |  | Body of pancreas |
|  | C252 |  | Tail of pancreas |
|  | C253 |  | Pancreatic duct |
|  | C257 |  | Other parts of pancreas |
|  | C258 |  | Overlapping lesion of pancreas |
|  | C259 |  | Pancreas, unspecified |
| K85 | K85 | Acute pancreatitis | Acute pancreatitis |
|  | K850 |  | Idiopathic acute pancreatitis |
|  | K851 |  | Biliary acute pancreatitis |
|  | K852 |  | Alcohol-induced acute pancreatitis |
|  | K858 |  | Other acute pancreatitis |
|  | K859 |  | Acute pancreatitis, unspecified |
| K86 | K860 | Other diseases of pancreas | Alcohol-induced chronic pancreatitis |
|  | K861 |  | Other chronic pancreatitis |
|  | K862 |  | Cyst of pancreas |
|  | K863 |  | Pseudocyst of pancreas |
|  | K868 |  | Other specified diseases of pancreas |
|  | K869 |  | Disease of pancreas, unspecified |
| Q45 | Q453 | Other congenital malformations of digestive system | Other congenital malformations of pancreas and pancreatic duct |
| D13 | D136 | Benign neoplasm of other and ill-defined parts of digestive system | Pancreas |

Figure S1: Vioplot of IPFD grouped by the history of pancreatic diseases


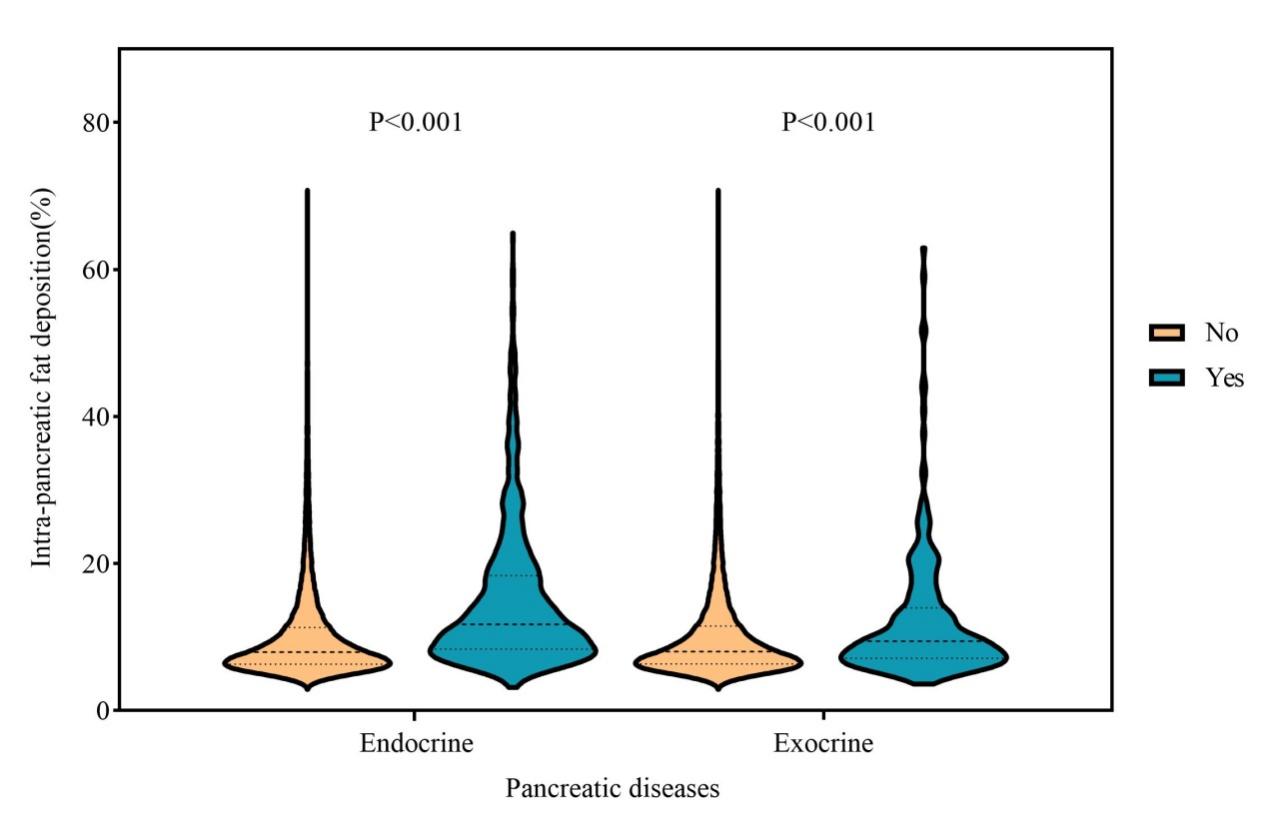


IPFD: Intra-pancreatic fat deposition.

Table S3: Impact of the five pancreatic endocrine disorders on IPFD separately

| Coding (ICD10) | Meaning | N | t | β (95%CI) | P |
| --- | --- | --- | --- | --- | --- |
| E10 | Insulin-dependent diabetes mellitus | 189 | -1.39 | -0.62 (-1.48, 0.25) | 0.165 |
| E11 | Non-insulin-dependent diabetes mellitus | 1951 | 14.98 | 2.15 (1.87, 2.43) | <0.001 |
| E13 | Other specified diabetes mellitus | 6 | 1.06 | 2.63 (-2.23, 7.49) | 0.290 |
| E14 | Unspecified diabetes mellitus | 225 | 3.08 | 1.25 (0.46, 2.05) | 0.002 |
| E16 | Other disorders of pancreatic internal secretion | 49 | -1.68 | -1.46 (-3.16, 0.24) | 0.093 |

Regression coefficient (β) was calculated from multiple linear regression models adjusted for age, sex, ethnic background, BMI, smoking status, alcohol drinker status, time spent watching television (TV), sleep duration, summed MET minutes per week for all activity and dyslipidemia.

IPFD: Intra-pancreatic fat deposition; BMI: Body mass index; MET: Metabolic equivalent task.

Table S4: The baseline characteristics of participants (sensitivity analysis)

| Characteristics | Overall | Pancreatic endocrine diseases | | P | Pancreatic exocrine diseases | | P |
| --- | --- | --- | --- | --- | --- | --- | --- |
|  | (N=47292) | No(N=45747) | Yes(N=1545) |  | No(N=47095) | Yes(N=197) |  |
| Age | 65.00 [59.00, 70.00] | 65.00 [59.00, 70.00] | 69.00 [63.00, 73.00] | <0.001 | 65.00 [59.00, 70.00] | 68.00 [63.00, 74.00] | <0.001 |
| Sex |  |  |  | <0.001 |  |  | 0.353 |
| Female | 23526 (49.7) | 23001 (50.3) | 525 (34.0) |  | 23435 (49.8) | 91 (46.2) |  |
| Male | 23766 (50.3) | 22746 (49.7) | 1020 (66.0) |  | 23660 (50.2) | 106 (53.8) |  |
| Ethnic background |  |  |  | <0.001 |  |  | 0.817 |
| Others | 1455 ( 3.1) | 1338 ( 2.9) | 117 ( 7.6) |  | 1450 ( 3.1) | 5 ( 2.5) |  |
| White | 45837 (96.9) | 44409 (97.1) | 1428 (92.4) |  | 45645 (96.9) | 192 (97.5) |  |
| BMI | 25.92 [23.53, 28.84] | 25.85 [23.48, 28.72] | 28.72 [25.64, 32.35] | <0.001 | 25.92 [23.53, 28.84] | 26.66 [24.16, 30.60] | 0.002 |
| Smoking |  |  |  | <0.001 |  |  | 0.866 |
| Never | 29658 (62.7) | 28839 (63.0) | 819 (53.0) |  | 29538 (62.7) | 120 (60.9) |  |
| Previous | 16097 (34.0) | 15428 (33.7) | 669 (43.3) |  | 16027 (34.0) | 70 (35.5) |  |
| Current | 1537 ( 3.3) | 1480 ( 3.2) | 57 ( 3.7) |  | 1530 ( 3.2) | 7 ( 3.6) |  |
| Alcohol |  |  |  | <0.001 |  |  | <0.001 |
| Never | 1454 ( 3.1) | 1367 ( 3.0) | 87 ( 5.6) |  | 1441 ( 3.1) | 13 ( 6.6) |  |
| Previous | 1645 ( 3.5) | 1550 ( 3.4) | 95 ( 6.1) |  | 1623 ( 3.4) | 22 (11.2) |  |
| Current | 44193 (93.4) | 42830 (93.6) | 1363 (88.2) |  | 44031 (93.5) | 162 (82.2) |  |
| Time spent watching television (TV) | 3.00 [2.00, 4.00] | 3.00 [2.00, 4.00] | 3.00 [2.00, 5.00] | <0.001 | 3.00 [2.00, 4.00] | 3.00 [2.00, 4.00] | 0.001 |
| Sleep duration | 7.00 [7.00, 8.00] | 7.00 [7.00, 8.00] | 7.00 [6.00, 8.00] | 0.310 | 7.00 [7.00, 8.00] | 7.00 [7.00, 8.00] | 0.584 |
| Summed MET minutes per week for all activity | 2217.00 [1139.00, 3933.00] | 2226.00 [1155.00, 3946.50] | 1695.00 [780.00, 3436.00] | <0.001 | 2217.00 [1140.00, 3933.00] | 2346.00 [1055.00, 3786.00] | 0.888 |
| Dyslipidemia |  |  |  | <0.001 |  |  | <0.001 |
| No | 43789 (92.6) | 42755 (93.5) | 1034 (66.9) |  | 43626 (92.6) | 163 (82.7) |  |
| Yes | 3503 ( 7.4) | 2992 ( 6.5) | 511 (33.1) |  | 3469 ( 7.4) | 34 (17.3) |  |
| IPFD | 7.93 [6.29, 11.32] | 7.85 [6.26, 11.12] | 11.57 [8.20, 17.99] | <0.001 | 7.93 [6.29, 11.31] | 9.01 [6.84, 13.82] | <0.001 |

Continuous values were presented as median (interquartile range) and categorical variables were presented as counts (percentages).

BMI: Body mass index; MET: Metabolic equivalent task; IPFD: Intra-pancreatic fat deposition.

Figure S2: Vioplot of IPFD grouped by the history of pancreatic diseases (sensitivity analysis)


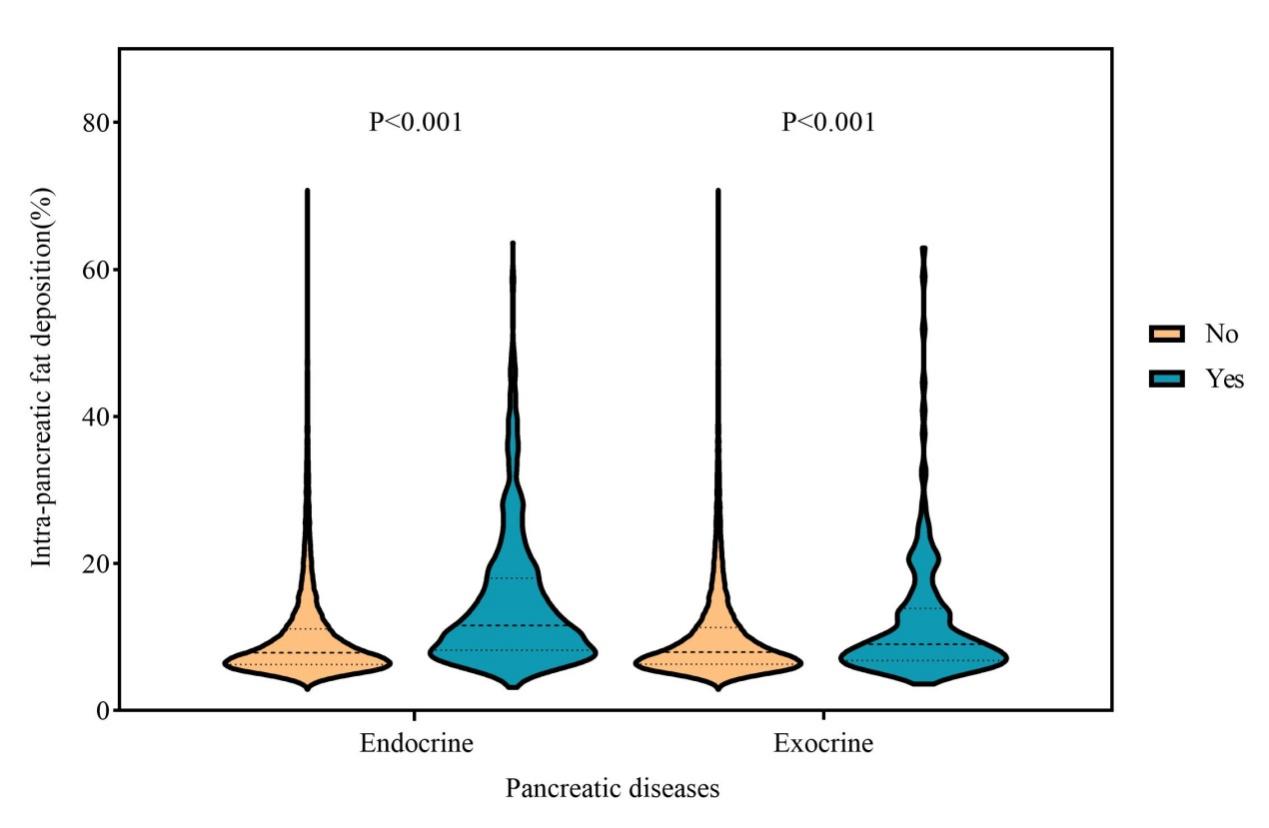


IPFD: Intra-pancreatic fat deposition.

Table S5: The extent to which pancreatic diseases alone and in combination with traditional independent variables, lifestyle habits and dyslipidemia affect IPFD (sensitivity analysis)

| Models | Pancreatic endocrine diseases | |  | Pancreatic exocrine diseases | |
| --- | --- | --- | --- | --- | --- |
|  | β (95%CI) | P |  | β (95%CI) | P |
| Model 0 | 4.67 (4.33, 5.02) | <0.001 |  | 1.86 (0.91, 2.81) | <0.001 |
| Model 1 | 2.04 (1.74, 2.35) | <0.001 |  | 0.49 (-0.34, 1.33) | 0.249 |
| Model 2 | 1.94 (1.63, 2.24) | <0.001 |  | 0.43 (-0.40, 1.27) | 0.311 |
| Model 3 | 1.79 (1.48, 2.10) | <0.001 |  | 0.37 (-0.46, 1.21) | 0.380 |

Model 0: unadjusted covariates.

Model 1: adjusted for age, sex, ethnic background and BMI.

Model 2: adjusted for age, sex, ethnic background, BMI, smoking status, alcohol drinker status, time spent watching television (TV), sleep duration and summed MET minutes per week for all activity.

Model 3: adjusted for age, sex, ethnic background, BMI, smoking status, alcohol drinker status, time spent watching television (TV), sleep duration, summed MET minutes per week for all activity and dyslipidemia.

IPFD: Intra-pancreatic fat deposition; BMI: Body mass index; MET: Metabolic equivalent task.

Table S6: Impact of the five pancreatic endocrine disorders on IPFD separately (sensitivity analysis)

| Coding (ICD10) | Meaning | N | t | β (95%CI) | P |
| --- | --- | --- | --- | --- | --- |
| E10 | Insulin-dependent diabetes mellitus | 153 | -0.62 | -0.30 (-1.25, 0.65) | 0.535 |
| E11 | Non-insulin-dependent diabetes mellitus | 1392 | 12.35 | 2.06 (1.73, 2.38) | <0.001 |
| E13 | Other specified diabetes mellitus | 4 | 0.77 | 2.28 (-3.56, 8.13) | 0.444 |
| E14 | Unspecified diabetes mellitus | 168 | 3.11 | 1.44 (0.53, 2.35) | 0.002 |
| E16 | Other disorders of pancreatic internal secretion | 33 | -1.34 | -1.39 (-3.43, 0.65) | 0.181 |

Regression coefficient (β) was calculated from multiple linear regression models adjusted for age, sex, ethnic background, BMI, smoking status, alcohol drinker status, time spent watching television (TV), sleep duration, summed MET minutes per week for all activity and dyslipidemia.

IPFD: Intra-pancreatic fat deposition; BMI: Body mass index; MET: Metabolic equivalent task.

Figure S3: Mediation models (sensitivity analysis)


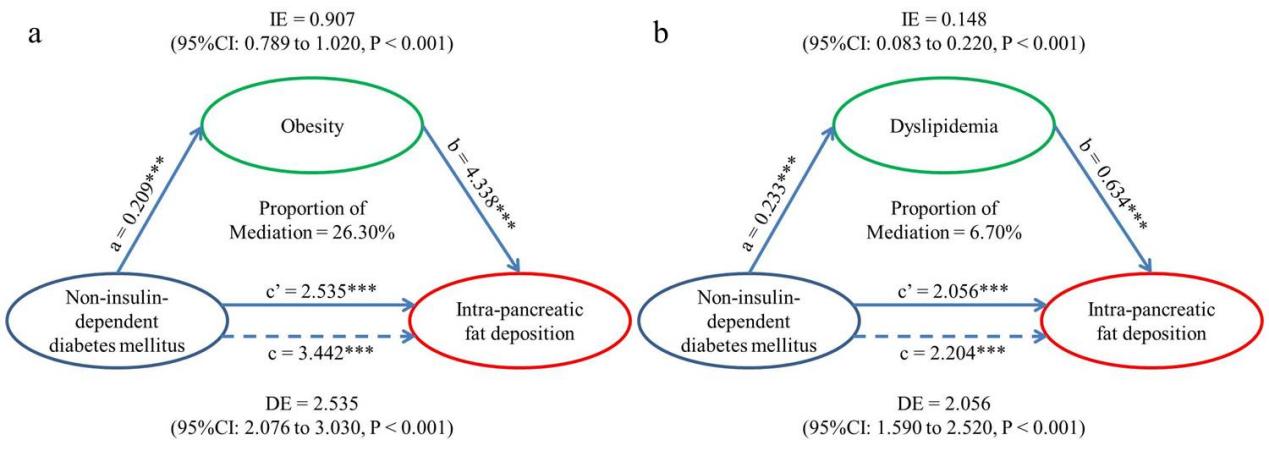


Figure S3a: Indirect effect (0.907; P < 0.001) of T2DM (exposure) towards IPFD (outcome) which was transmitted through obesity (mediator). Direct effect (2.535; P < 0.001) of T2DM (exposure) towards IPFD (outcome) which was the residual influence after accounting for obesity (mediator). Total effect (3.442; P < 0.001) of T2DM (exposure) towards IPFD (outcome) without considering the effect of obesity.

Figure S3b: Indirect effect (0.148; P < 0.001) of T2DM (exposure) towards IPFD (outcome) which was transmitted through dyslipidemia (mediator). Direct effect (2.056; P < 0.001) of T2DM (exposure) towards IPFD (outcome) which was the residual influence after accounting for dyslipidemia (mediator). Total effect (2.204; P < 0.001) of T2DM (exposure) towards IPFD (outcome) without considering the effect of dyslipidemia.

IPFD: Intra-pancreatic fat deposition; IE: Indirect effect, equivalent to a*b; DE: Direct effect, equivalent to c’; TE: Total effect, equivalent to c.

***, P < 0.001
