# Supplementary material for: The association between pancreatic diseases and pancreatic fat content: a cross-sectional study from the UK Biobank
Source: Front Endocrinol (Lausanne). 2025 Jun 6;16:1591652. doi: 10.3389/fendo.2025.1591652 (PMC12178842; doi:10.3389/fendo.2025.1591652)
Supplement: Supplementary file 2 [file Table1.docx]

Table 1: The baseline characteristics of participants

| Characteristics | Overall | Pancreatic endocrine diseases | | P | Pancreatic exocrine diseases | | P |
| --- | --- | --- | --- | --- | --- | --- | --- |
|  | (N=61088) | No(N=58939) | Yes(N=2149) |  | No(N=60823) | Yes(N=265) |  |
| Age | 66.00 [59.00, 71.00] | 65.00 [59.00, 71.00] | 70.00 [64.00, 74.00] | <0.001 | 66.00 [59.00, 71.00] | 69.00 [63.00, 74.00] | <0.001 |
| Sex |  |  |  | <0.001 |  |  | 0.331 |
| Female | 31672 (51.8) | 30914 (52.5) | 758 (35.3) |  | 31543 (51.9) | 129 (48.7) |  |
| Male | 29416 (48.2) | 28025 (47.5) | 1391 (64.7) |  | 29280 (48.1) | 136 (51.3) |  |
| Ethnic background |  |  |  | <0.001 |  |  | 0.986 |
| Others | 1971 ( 3.2) | 1795 ( 3.0) | 176 ( 8.2) |  | 1963 ( 3.2) | 8 ( 3.0) |  |
| White | 59117 (96.8) | 57144 (97.0) | 1973 (91.8) |  | 58860 (96.8) | 257 (97.0) |  |
| BMI | 25.99 [23.54, 28.95] | 25.91 [23.49, 28.82] | 28.78 [25.66, 32.32] | <0.001 | 25.98 [23.54, 28.94] | 27.26 [24.38, 31.05] | <0.001 |
| Smoking |  |  |  | <0.001 |  |  | 0.704 |
| Never | 38195 (62.5) | 37083 (62.9) | 1112 (51.7) |  | 38035 (62.5) | 160 (60.4) |  |
| Previous | 20890 (34.2) | 19935 (33.8) | 955 (44.4) |  | 20793 (34.2) | 97 (36.6) |  |
| Current | 2003 ( 3.3) | 1921 ( 3.3) | 82 ( 3.8) |  | 1995 ( 3.3) | 8 ( 3.0) |  |
| Alcohol |  |  |  | <0.001 |  |  | <0.001 |
| Never | 2040 ( 3.3) | 1909 ( 3.2) | 131 ( 6.1) |  | 2020 ( 3.3) | 20 ( 7.5) |  |
| Previous | 2246 ( 3.7) | 2106 ( 3.6) | 140 ( 6.5) |  | 2218 ( 3.6) | 28 (10.6) |  |
| Current | 56802 (93.0) | 54924 (93.2) | 1878 (87.4) |  | 56585 (93.0) | 217 (81.9) |  |
| Time spent watching television (TV) | 3.00 [2.00, 4.00] | 3.00 [2.00, 4.00] | 3.00 [2.00, 5.00] | <0.001 | 3.00 [2.00, 4.00] | 3.00 [2.00, 4.00] | <0.001 |
| Sleep duration | 7.00 [7.00, 8.00] | 7.00 [7.00, 8.00] | 7.00 [6.00, 8.00] | 0.239 | 7.00 [7.00, 8.00] | 7.00 [7.00, 8.00] | 0.559 |
| Summed MET minutes per week for all activity | 2190.00 [1132.50, 3912.00] | 2213.00 [1150.00, 3932.00] | 1704.00 [792.00, 3439.50] | <0.001 | 2190.00 [1133.00, 3916.25] | 2186.00 [924.00, 3732.00] | 0.193 |
| Dyslipidemia |  |  |  | <0.001 |  |  | <0.001 |
| No | 56282 (92.1) | 54876 (93.1) | 1406 (65.4) |  | 56067 (92.2) | 215 (81.1) |  |
| Yes | 4806 ( 7.9) | 4063 ( 6.9) | 743 (34.6) |  | 4756 ( 7.8) | 50 (18.9) |  |
| IPFD | 8.03 [6.34, 11.49] | 7.94 [6.30, 11.29] | 11.72 [8.39, 18.32] | <0.001 | 8.03 [6.33, 11.48] | 9.44 [7.09, 13.94] | <0.001 |

Continuous values were presented as median (interquartile range) and categorical variables were presented as counts (percentages).

BMI: Body mass index; MET: Metabolic equivalent task; IPFD: Intra-pancreatic fat deposition.
