# Supplementary material for: The association between pancreatic diseases and pancreatic fat content: a cross-sectional study from the UK Biobank
Source: Front Endocrinol (Lausanne). 2025 Jun 6;16:1591652. doi: 10.3389/fendo.2025.1591652 (PMC12178842; doi:10.3389/fendo.2025.1591652)
Supplement: Supplementary file 3 [file Table2.docx]

Table 2: The extent to which pancreatic diseases alone and in combination with traditional independent variables, lifestyle habits and dyslipidemia affect IPFD

| Models | Pancreatic endocrine diseases | |  | Pancreatic exocrine diseases | |
| --- | --- | --- | --- | --- | --- |
|  | β (95%CI) | P |  | β (95%CI) | P |
| Model 0 | 4.76 (4.47, 5.06) | <0.001 |  | 1.89 (1.05, 2.72) | <0.001 |
| Model 1 | 2.13 (1.86, 2.39) | <0.001 |  | 0.36 (-0.37, 1.10) | 0.334 |
| Model 2 | 2.02 (1.76, 2.29) | <0.001 |  | 0.27 (-0.47, 1.00) | 0.478 |
| Model 3 | 1.86 (1.60, 2.13) | <0.001 |  | 0.20 (-0.54, 0.93) | 0.599 |

Model 0: unadjusted covariates.

Model 1: adjusted for age, sex, ethnic background and BMI.

Model 2: adjusted for age, sex, ethnic background, BMI, smoking status, alcohol drinker status, time spent watching television (TV), sleep duration and summed MET minutes per week for all activity.

Model 3: adjusted for age, sex, ethnic background, BMI, smoking status, alcohol drinker status, time spent watching television (TV), sleep duration, summed MET minutes per week for all activity and dyslipidemia.

IPFD: Intra-pancreatic fat deposition; BMI: Body mass index; MET: Metabolic equivalent task.
